# Atypical presentation of colon adenocarcinoma: a case report

**DOI:** 10.1186/1752-1947-6-58

**Published:** 2012-02-13

**Authors:** Lynnette K Tumwine, Magid Kagimu, Ponsiano Ocama, Innocent Segamwenge, Noah Masiira-Mukasa, Dan Wamala, Otto Dworak, Christopher K Opio

**Affiliations:** 1 Department of Pathology, School of Biomedical Sciences, College of Health Sciences, Makerere University, P.O.Box 7072, Kampala, Uganda; 2 Department of Medicine, School of Medicine, College of Health Sciences, Makerere University, P.O.Box 7072, Kampala, Uganda; 3 Department of Surgery, School of Medicine, College of Health Sciences, Makerere University, P.O.Box 7072, Kampala, Uganda; 4 Fuerth Teaching Hospital, University of Erlangen, Fuerth, Germany

## Abstract

**Introduction:**

Adenocarcinoma of the colon is the most common histopathological type of colorectal cancer. In Western Europe and the United States, it is the third most common type and accounts for 98% of cancers of the large intestine. In Uganda, as elsewhere in Africa, the majority of patients are elderly (at least 60 years old). However, more recently, it has been observed that younger patients (less than 40 years of age) are presenting with the disease. There is also an increase in its incidence and most patients present late, possibly because of the lack of a comprehensive national screening and preventive health-care program. We describe the clinicopathological features of colorectal carcinoma in the case of a young man in Kampala, Uganda.

**Case presentation:**

A 27-year-old man from Kampala, Uganda, presented with gross abdominal distension, progressive loss of weight, and fever. He was initially screened for tuberculosis, hepatitis, and lymphoma, and human immunodeficiency virus/acquired immunodeficiency syndrome infection. After a battery of tests, a diagnosis of colorectal carcinoma was finally established with hematoxylin and eosin staining of a cell block made from the sediment of a liter of cytospun ascitic fluid, which showed atypical glands floating in abundant extracellular mucin, suggestive of adenocarcinoma. Ancillary tests with alcian blue/periodic acid Schiff and mucicarmine staining revealed that it was a mucinous adenocarcinoma. Immunohistochemistry showed strong positivity with CDX2, confirming that the origin of the tumor was the colon.

**Conclusions:**

Colorectal carcinoma has been noted to occur with increasing frequency in young adults in Africa. Most patients have mucinous adenocarcinoma, present late, and have rapid disease progression and poor outcome. Therefore, colorectal malignancy should no longer be excluded from consideration only on the basis of a patient's age. A high index of suspicion is important in the diagnosis of colorectal malignancy in young African patients.

## Introduction

Adenocarcinoma of the colon is the most common histopathological type of colorectal carcinoma. It ranks fourth in men and third in women in Western Europe and the US and overall accounts for 98% of cancers of the large intestine [1]. This tumor has been largely associated with a 'Western' lifestyle (obesity, lack of physical activity, consumption of diets low in fruit and vegetables, and overconsumption of red meat), hence its predominance in affluent societies. In addition to lifestyle, pre-malignant conditions such as familial adenomatous polyposis coli syndrome and inflammatory bowel disease are important associated factors [2].

In sub-Saharan Africa, evidence shows that the incidence of adenocarcinoma of the colon is rising and this has been attributed to the change in lifestyle as a result of globalization [1,3]. In Uganda, the Kampala Cancer Registry in the Department of Pathology, School of Biomedical Sciences, College of Health Sciences, Makerere University, which covers a population of two million people in Kyadondo County, has shown an increased trend in the past three decades. In the period from 1960 to 1997, incidences have increased from three to 6.8 per 100,000 in women and from 2.7 to 6.6 per 100,000 in men [3]. This trend has been observed in other low-income countries where the incidence was once low [4].

The low incidence in Africans as compared with Caucasians results from consumption of diets rich in fiber, which is a common practice, and the rarity of the pre-malignant familial polyposis syndromes and inflammatory bowel disease (ulcerative colitis and Crohn's disease) [2]. However, urbanization and civilization have led to changes in dietary habits and to less exercise. This might be the reason for this rising trend.

Diagnostic workup is largely in line with the presenting features of the patients, and the diagnosis is usually made on the basis of colonoscopy and biopsy. However, when the presentation is unusual (as in our patient), diagnosis is difficult. We describe an atypical presentation of adenocarcinoma of the colon in a young man and discuss the challenges of diagnosis in a resource-limited setting.

## Case presentation

A 27-year-old man from Kampala, Uganda, presented with a three-month history of progressive abdominal swelling and discomfort to our hospital three years ago. He was well until three months prior to admission; he developed fever, malaise, and drenching night sweats and noticed a progressive loss of weight. He did not have vomiting, diarrhea, or yellow eyes. An examination of his other systems revealed no other relevant findings. His medical and surgical history was unremarkable. He was a student and did not consume alcohol or smoke.

During an examination, he was sick-looking and wasted and had a body mass index of 20. He had no lymphadenopathy or stigmata of liver disease, human immunodeficiency virus/acquired immunodeficiency syndrome (HIV/AIDS), lymphoma, or any mucocutaneous abnormalities. The results of an abdominal examination were remarkable for ascites as evidenced by shifting dullness. The results of a rectal examination were reported as normal. The results of the rest of his physical examination were also normal. He was worked up for his ascites and wasting.

Routine diagnostic paracentesis and evaluation of the ascitic fluid were done while our patient was awaiting ultrasonography. A straw-colored, slightly blood-stained fluid showed total protein of 4.2 g/dL, glucose of 80 mg/dL, total white cell count of 30,000 cells/mm^3^, white cell differential counts were as follows: neutrophils 24% and lymphocytes 76%. Gram and Ziehl-Neelsen stains were negative, the serum-ascites-albumin gradient was less than 1.1, and ascitic protein was more than 2.5 g/dL. These findings pointed toward a peritoneal pathology, probably malignancy of the peritoneum.

An abdominal ultrasound confirmed gross ascites. The peritoneum was markedly thickened, nodular, and irregular with areas of cystic change and involvement of the omentum. The liver, spleen, and kidneys appeared normal. There were no features of enlarged lymph nodes.

A barium meal examination showed a transverse ulcer in the sigmoid colon and thickened loops. Later, a lower gastrointestinal tract flexible sigmoidoscopy showed an extrinsic mass protruding from the anterior wall of the rectum, which was irregular during a rectal examination. A large polyp was seen in the sigmoid colon and a biopsy was taken and sent for histopathology.

A chest X-ray was normal. A hematological evaluation was normal except for a mild thrombocytosis of 550,000/mm^3 ^and an erythrocyte sedimentation rate of 90 mm/hour. The results of serum biochemical tests were normal. Screenings for HIV and hepatitis B were negative. The results of a routine stool examination were also normal.

**Figure 1:**
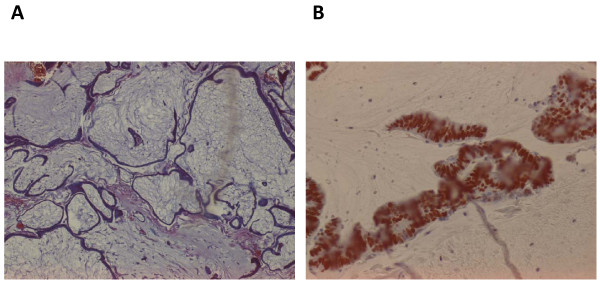
**(**A**) Hematoxylin and eosin staining of cell block shows a colloid carcinoma with malignant mucin-filled epithelial cells floating free in mucinous pools**. (**B**) Immunohistochemistry staining using CDX2 is strongly positive in colorectal carcinoma.

**Figure 2:**
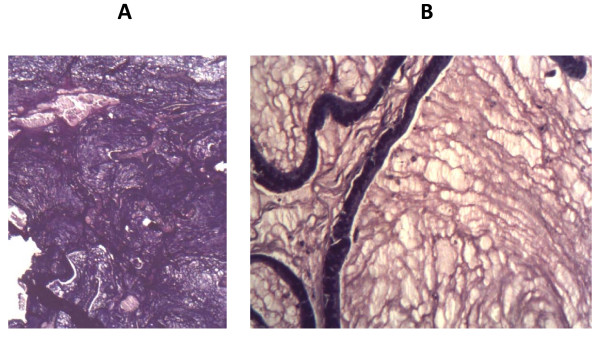
**(**A**) Mucicarmine staining shows epithelial strips floating in mucin, which stains red**. (**B**) Alcian blue/periodic acid Schiff stains neutral mucin magenta.

A histopathological analysis of cell blocks made from the sediment of cytospun ascitic fluid and a sigmoid colon polyp obtained at sigmoidoscopy revealed atypical, malignant, deeply basophilic epithelial glands suggestive of adenocarcinoma (Figure 1A). An alcian blue/periodic acid Schiff and mucicarmine staining was positive for neutral mucin, which stained magenta, confirming mucinous adenocarcinoma (Figure 2A, B).

For staging purposes, abdominal computed tomography (CT) was done. Pre- and post-contrast axial CT scans of the abdomen were done at 7 mm-thick slices. They showed extensive multiloculated hypodense cystic areas in the peritoneal cavity with the largest loculi measuring 152 × 123 × 263 mm. The liver was enlarged but was free of focal masses. The spleen, kidneys, and urinary bladder were normal. Some of the intestinal loops were thickened. The retroperitoneal areas appeared normal. There were features of extensive multiloculated ascites with hepatomegally. No other abdominal masses were seen.

Cytoreductive abdominal surgery revealed a bulky copious mucinous gelatinous tumor filling the abdominal cavity making detailed examination and resection difficult. Therefore, a sample was taken for cytopathological examination and it corroborated the previous finding of mucinous adenocarcinoma. Immunohistochemistry was done at the Fuerth Teaching Hospital at the University of Erlangen (Germany). Staining with CDX2 showed strong positivity, confirming our suspicion of a primary colon tumor (Figure 1B).

Our patient had initially received a two-week therapeutic trial for tuberculosis with a drug regimen of rifampicin, isoniazid, ethambutol, and pyrazinamide. This treatment was discontinued when the diagnosis of adenocarcinoma was made.

Tumor staging revealed advanced disease and palliative care, which included counseling, frequent abdominocentesis and pain management with oral morphine, was given. Our patient died of the disease.

**Figure 3:**
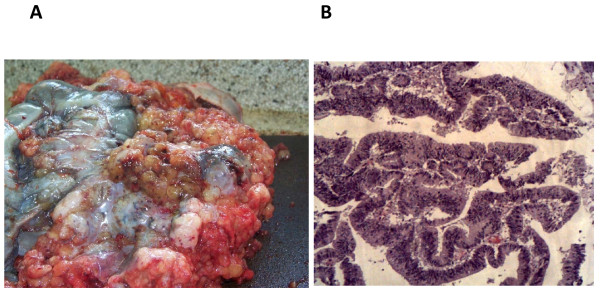
**(**A**) At autopsy, mucinous gelatinous polypoid masses arising from the sigmoid colon and covering the serosa are seen**. (**B**) Hematoxylin and eosin staining of the masses in (A) showed an invasive well-differentiated adenocarcinoma.

During the autopsy, a general examination revealed severe cachexia and marked abdominal distension. After a midline abdominal incision was made, a mucinous gelatinous hemorrhagic tumor surrounding all of the abdominal organs and infiltrating the diaphragm was seen. On closer examination, the tumor was seen to be emanating from the sigmoid colon and was tightly adherent to the rest of the colon and other abdominal viscera (Figure 3A). Further colonic dissection revealed multiple ulcerated and necrotic polyps 2 mm to 3 mm in diameter in the sigmoid colon. A histopathological examination of these polyps revealed a pseudostratified glandular epithelium with marked cellular atypia and a papillary pattern (Figure 3B).

## Discussion

Adenocarcinoma of the colon in young adults in sub-Saharan Africa is a perceived rarity [5]. However, increasing reports show that it occurs often [6]. Rubin and colleagues [5] reported a series of three young adults from Eastern Africa (two from Kenya and one from Ethiopia) who had peculiar presentations similar to those of our patient. The three lived in the US for a period of six to 18 years before disease presentation, and, as with our patient, two had a family history of colon cancer or inflammatory bowel disease [5]. However, unlike our patient, who had none of the symptomatology of colon cancer, one of the patients presented with malena, and the others presented with frank rectal bleeding, abdominal pain, and nausea [5].

A study in Turkey revealed the occurrence of colorectal carcinoma in children younger than 18 years of age. These children all presented with advanced disease (Dukes stage C and D) and had peritoneal involvement while the predominant histopathological type was mucinous adenocarcinoma, as was the case in our patient [7].

Peritoneal carcinomatosis is estimated to occur in about one out of 10 individuals (10%) with colorectal carcinoma [8]. Intraperitoneal spread in colorectal carcinoma results from full-thickness invasion of the bowel wall by an invasive tumor or as a result of the rupture of a structure by a non-invasive tumor, such as the mucus-producing cystadenocarcinoma of the appendix. In our patient, the former was more likely. Most colorectal carcinomas occur after malignant transformation of adenomatous polyps. Transformation involves inactivation of tumor suppressor genes (adenomatous polyposis coli, p53 genes), mutation of oncogenes and or growth regulators (K-ras), and mutations caused by dysfunction in the deoxyribonucleic acid (DNA) mismatch repair genes (MMR genes) [9].

Colonoscopy and postmortem examination revealed that our patient had multiple polyps. This might explain why he developed adenocarcinoma of the colon. It is likely, given his young age and the presence of multiple colon polyps, that he had one of the polyposis coli syndromes [10]. We did not undertake genetic studies to corroborate this.

Our patient presented with 'B' symptoms (fever, drenching sweats, and more than 10% weight loss) and ascites. The differential diagnosis for this presentation in our setting includes HIV/AIDS with its comorbidities, decompensated liver disease, abdominal tuberculosis, tumors of lymphoid origin, and peritoneal carcinomatosis [11]. All of these were excluded after thorough investigations were done.

Several studies have shown that routine cytopathology using ascitic fluid smears is positive for malignant cells in two out of 10 patients with ascites [12]. This is because, unless the ascitic fluid is cytospun, smears on slides will give false-negative results since the malignant cells are widely dispersed in the vast amounts of ascitic fluid. Often, clinicians submit only tiny amounts (5 to 10 mL) of fluid, which are not diagnostically useful.

Large amounts of ascitic fluid (at least 500 mL) should be sent to the pathology laboratories and cytospun so that the sediment is made into cell blocks. This is the best way of detecting malignancy since the malignant cells will sediment at the bottom and will be available after the rest of the fluid is decanted away to make it into a cell block. Cytopathology, if properly carried out, has a reported sensitivity of 60% and a specificity of 100% [13].

The histopathological types of colorectal carcinoma include adenocarcinoma, which is the most common type [14]. Adenocarcinomas may be well-differentiated, often arising within a villous adenoma, or poorly-differentiated. The poorly-differentiated tumors (for example, signet ring cell carcinomas) have a poor prognosis and tend to affect younger patients. Most are well-differentiated adenocarcinomas and are classified according to mucin content. Mucin-secreting adenocarcinomas have less than 50% mucin production, mucinous carcinomas have more than 50% extracellular mucin, and signet ring carcinomas have intracellular mucin that displaces the nucleus to one side [15]. Diagnosis of signet ring adenocarcinomas is made when at least 50% of the cells are of the signet ring type [16]. Young people most commonly present with advanced disease and have defects in DNA MMR and microsatellite instability [10].

The other histological variants of colorectal carcinomas are squamous cell carcinoma, adenosquamous carcinoma, malignant carcinoid tumors, and embryonal rhabdomyosarcomas, the last of which are very rare. The rest are undifferentiated and medullary adenocarcinomas [17].

## Conclusions

Colorectal carcinoma has been observed to occur with increasing frequency in young adults in Africa. Most patients have mucinous adenocarcinoma, present late, progress rapidly, and have a poor outcome. Therefore, colorectal malignancy should no longer be excluded only on the basis of the patient's age. A high index of suspicion is important in the diagnosis in African patients younger than 40 years of age.

## Consent

Written informed consent was obtained from the patient's next-of-kin for publication of this case report and any accompanying images. A copy of the written consent is available for review by the Editor-in-Chief of this journal.

## Competing interests

The authors declare that they have no competing interests.

## Authors' contributions

LKT conceived the idea, made the preliminary diagnosis of colorectal carcinoma, and wrote the manuscript. IS, MK, PO, and NM-M admitted and treated the patient. DW confirmed the diagnosis of colorectal carcinoma and established the collaboration with Fuerth Teaching Hospital for immunohistochemistry. OD interpreted the immunocytochemistry. KCO treated the patient and helped to write the manuscript. All authors read and approved the final manuscript.
